# A prognostic nomogram for overall survival in male breast cancer with histology of infiltrating duct carcinoma after surgery

**DOI:** 10.7717/peerj.7837

**Published:** 2019-10-14

**Authors:** Xin Chai, Mei-yang Sun, Hong-yao Jia, Min Wang, Ling Cao, Zhi-wen Li, Dun-wei Wang

**Affiliations:** 1 Breast Surgery Department, Jilin Provincial Cancer Hospital, Changchun, Jilin, China; 2 Breast Surgery Department, First Hospital of Jilin University, Changchun, Jilin, China; 3 Department of Pathology, Jilin Provincial Cancer Hospital, Changchun, Jilin, China; 4 Department of Radiation Oncology, Cancer Hospital of Jilin Province, Changchun, Jilin, China; 5 Department of Anesthesiology, First Hospital of Jilin University, Changchun, Jilin, China

**Keywords:** Nomogram, Overall survival, Prognosis, SEER database, Male breast cancer

## Abstract

**Objective:**

The study was designed to construct and validate a nomogram for predicting overall survival (OS) of male breast cancer (MBC) patients with infiltrating duct carcinoma (IDC).

**Methods:**

The cohort was selected from the Surveillance, Epidemiology, and End Results (SEER) database between January 1, 2004 and December 31, 2013. Univariate and multivariate Cox proportional hazard (PH) regression models were performed. A nomogram was developed based on the significant prognostic indicators of OS. The discriminatory and predictive capacities of nomogram were assessed by Harrell’s concordance index (C-index), calibration plots, area under the curve (AUC) and the decision curve analysis (DCA).

**Results:**

The median and maximal survival time of 1862 eligible patients were 49 and 131 months, respectively. Multivariate analysis showed that age (*P* < 0.0001), marital status (*P* = 0.002), T stage (*P* < 0.0001), N stage (*P* = 0.021), M stage (*P* < 0.0001), progesterone receptor (PR) (*P* = 0.046), human epidermal growth factor receptor-2 (HER2) (*P* = 0.009), and chemotherapy (*P* = 0.003) were independent prognostic indicators of IDC of MBC. The eight variables were then combined to construct a 3-and 5-year nomogram. The C-indexes of the nomogram were0.740 (95% confidence interval [CI] [0.709–0.771]) and 0.718 (95% CI [0.672–0.764]) for the internal validation and external validation, respectively. A better discriminatory capacity was observed in the nomogram compared with the SEER summary stage (*P* < 0.001) and AJCC TNM staging systems (6th edition; *P* < 0.001) with respect to OS prediction. Good consistency was detected between the nomogram prediction and actual findings, as indicated by calibration curves. The AUC for 3-and 5-year OS was 0.739 (95% CI [0.693–0.786]) and 0.764 (95% CI [0.725–0.803]) in the training cohort and 0.737 (95% CI [0.671–0.803]) and 0.735 (95% CI [0.678–0.793]) in the validation cohort, respectively. The DCA demonstrated that the survival nomogram was clinically useful.

**Conclusions:**

The nomogram was able to more accurately predict 3-and 5-year OS of MBC patients with IDC histology than were existing models.

## Introduction

Male breast cancer (MBC) is a rare malignancy accounting for less than 1% of all male cancers and less than 1% of all patients with breast cancer (Korde et al., 2010). Moreover, MBC is responsible for no more than 0.2% of all cancer-associated mortality in males (Weiss, Moysich & Swede, 2005). Due to its rare incidence, MBC data are mainly acquired from small, single-centered, retrospective research or extrapolated from randomized prospective studies or clinical experience of female breast cancer (FBC) (Giordano, Buzdar & Hortobagyi, 2002).

TNM staging classification is a common tool for predicting the outcomes of patients with cancer by evaluation of tumor size and location (T), regional lymph node involvement (N), and distant metastasis (M) (Burke, 2004). However, TNM classification is not efficient enough to encompass cancer biology or predict the outcomes of breast cancer, especially for MBC (Park et al., 2011). Furthermore, other clinical factors such as age, race, tumor location, grade, adjuvant treatments, and molecular characteristics can all influence the prognosis of MBC patients (Yalaza, Inan & Bozer, 2016). The nomogram, a simple statistical predictive tool, has been shown to compare favorably with the traditional TNM staging systems in multiple types of cancers (Dai, Jin & Wang, 2018; Fang et al., 2017; Iasonos et al., 2008; Song et al., 2018; Sternberg, 2006).

Different histological subtypes show diverse prognoses in patients with breast cancer (McCready et al., 2000). Infiltrating duct carcinoma (IDC) accounts for over 90% of all MBC cases, and other pathological types are extremely rare (Cutuli, 2007; Fentiman, Fourquet & Hortobagyi, 2006). Therefore, the prediction of IDC type of MBC is relatively important. At present, no studies have specially constructed a nomogram for the overall survival (OS) of the IDC type of MBC.

Thus, the current study was designed to assess the prognostic value of clinicopathological characteristics of MBC patients with IDC histological type and to construct a nomogram for their prognostic prediction.

## Materials and Methods

### Ethics statement

The National Cancer Institute’s Surveillance, Epidemiology, and End Results (SEER) program uses population-based data to develop comprehensive sources, initiated from 1973 and annually updated (Duggan et al., 2016), covering approximately 30% of the US population of several different geographic regions (Cronin, Ries & Edwards, 2014). The SEER Research Data Agreement was signed to allow access to SEER information with the use of reference number 16462-Nov2016. We performed the research methods to obtain data following the approved guidelines. Afterward, the Office for Human Research Protection determined that the data analysis was of nonhuman subjects, who were researched by the United States Department of Health and Human Services, as they were publicly available and de-identified; therefore, no approval was required by the institutional review board.

### Study population

Patient data were obtained using the SEER database (Submission, November 2016). The SEER*State v8.3.5 tool, released on March 6, 2018, was used to determine and select eligible patients. Moreover, the study duration ranged from January 1, 2004 to December 31, 2013. The following inclusion criteria were used for data screening: (1) age at the diagnosis ≥20 years, (2) only primary MBC patients undergoing surgery were enrolled, and (3) the histological type should be IDC(ICD-O-3 Histology/behavior-8500/3). The exclusion criteria were listed as follows: (1) patients under 20 years old, (2) patients burdened with two or more primary malignancies, (3) patients with incomplete or inaccessible survival data, (4) patients only clinically diagnosed, (5) patients without important clinicopathological information, such as age at diagnosis, laterality, race, tumor location and size, grade, 6th AJCC tumor stage , SEER summary stage, ER, or PR situation, and (6) patients who did not receive surgery or died within 3 months after surgery. The remaining patients were enrolled as the SEER primary cohort. Among them, patients from eight randomly selected registries (Seattle, Louisiana, Utah, New Jersey, San Francisco-Oakland SMSA, San José-Monterey, Rural Georgia, and New Mexico) were defined as the validation cohort, while the others were considered the SEER training data cohort.

### Covariates and endpoint

The following demographic and clinical variables were obtained from the SEER dataset, including: age at diagnosis, laterality, marital status, primary tumor location and size, race, grade, T, N, and M stage, estrogen receptor (ER), progesterone receptor (PR), human epidermal growth factor receptor-2 (HER2), chemotherapy, radiotherapy, and follow-up information. The widowed, single (never married or having a domestic partner), divorced or separated patients were classified as unmarried. Continuous variables, including age and tumor size, were further transformed into categorical ones according to the recognized cutoff values. The 6th edition of AJCC TNM staging classification was utilized, and the population studied ranged from 2004 to 2013 because relevant data in the SEER dataset were published in 2004.

The endpoint of this study was OS, which was defined as the duration period from diagnosis to the most recent follow-up date or date of death. There was a predetermined cutoff date based on the SEER 2016 submission database, containing death information until 2014. Therefore, the cutoff date of December 31, 2014 was used.

### Statistical analysis

#### Nomogram construction

Baseline continuous and categorical variables were shown as median with range and numbers with proportions, respectively. In addition, the chi-square test or Fisher’s exact test were utilized for comparison. Cox proportional hazard (PH) regression model was used to calculate the hazard ratio (HR) along with the corresponding 95% confidence interval (CI) for each potential risk factor. Backward stepwise in Cox PH regression model resulted in the successful identification of all independent risk indicators. A nomogram model was constructed based on the training set data. The nomogram was established for predicting 3- and 5-year OS using the package of rms in R software version 3.51, which included all independent prognostic indicators. A two-sided *P* < 0.05 was considered statistically significant.

#### Nomogram validation

The nomogram was validated through the measurement of discrimination and calibration, both internally (training cohort) and externally (validation cohort). The concordance index (C-index), measuring the differences in predictive capacity between observed and predicted outcomes, was used to assess the discrimination of the nomogram (Wolbers et al., 2009). A higher C-index suggested a superior capacity to discriminate patients with diverse survival outcomes. Rcorrp.cens package in Hmisc in R was utilized for comparisons between nomogram and TNM staging or SEER summary stage, followed by the assessment of the C-index. Receiver operating characteristic (ROC) curves were also used to verify the nomogram score. The marginal estimate versus model was used to establish a calibration plot representing the calibration between nomogram-predicted and observed survival. A calibration plot along the 45° line implicated a perfect model, with great consistency between the predicted and actual outcomes. The clinical usefulness and benefits of the predictive model were estimated by decision curve analyses (DCA) (Vickers & Elkin, 2006).

SPSS software version 23(SPSS Inc., Chicago, USA) as well as the R software version 3.51 (R Core Team, 2018) were used for statistical analysis. A *P* < 0.05 was considered statistically significance.

## Results

### Patient screening process

**Figure 1:**
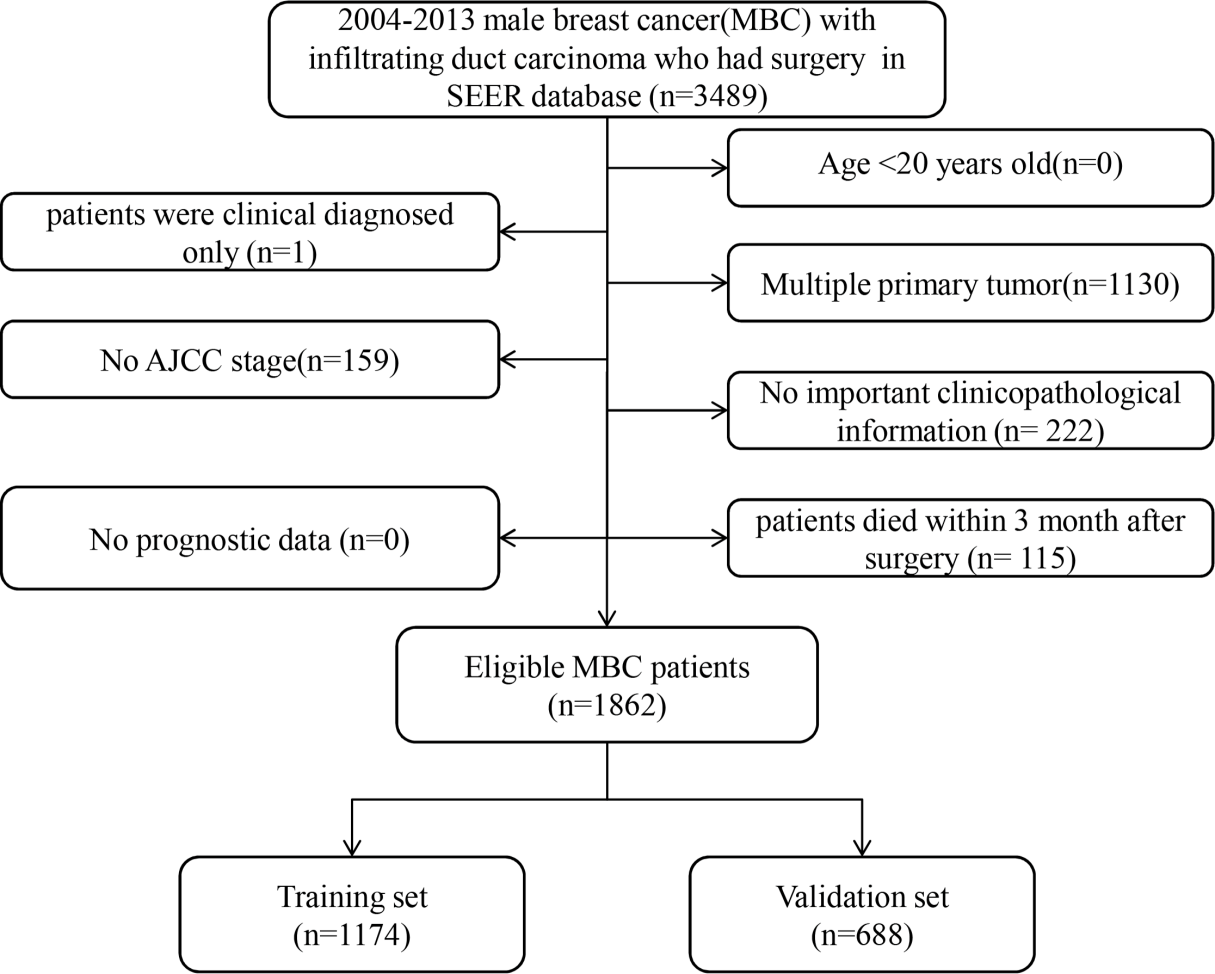
Flow chart for screening eligible patients.

**Figure 2:**
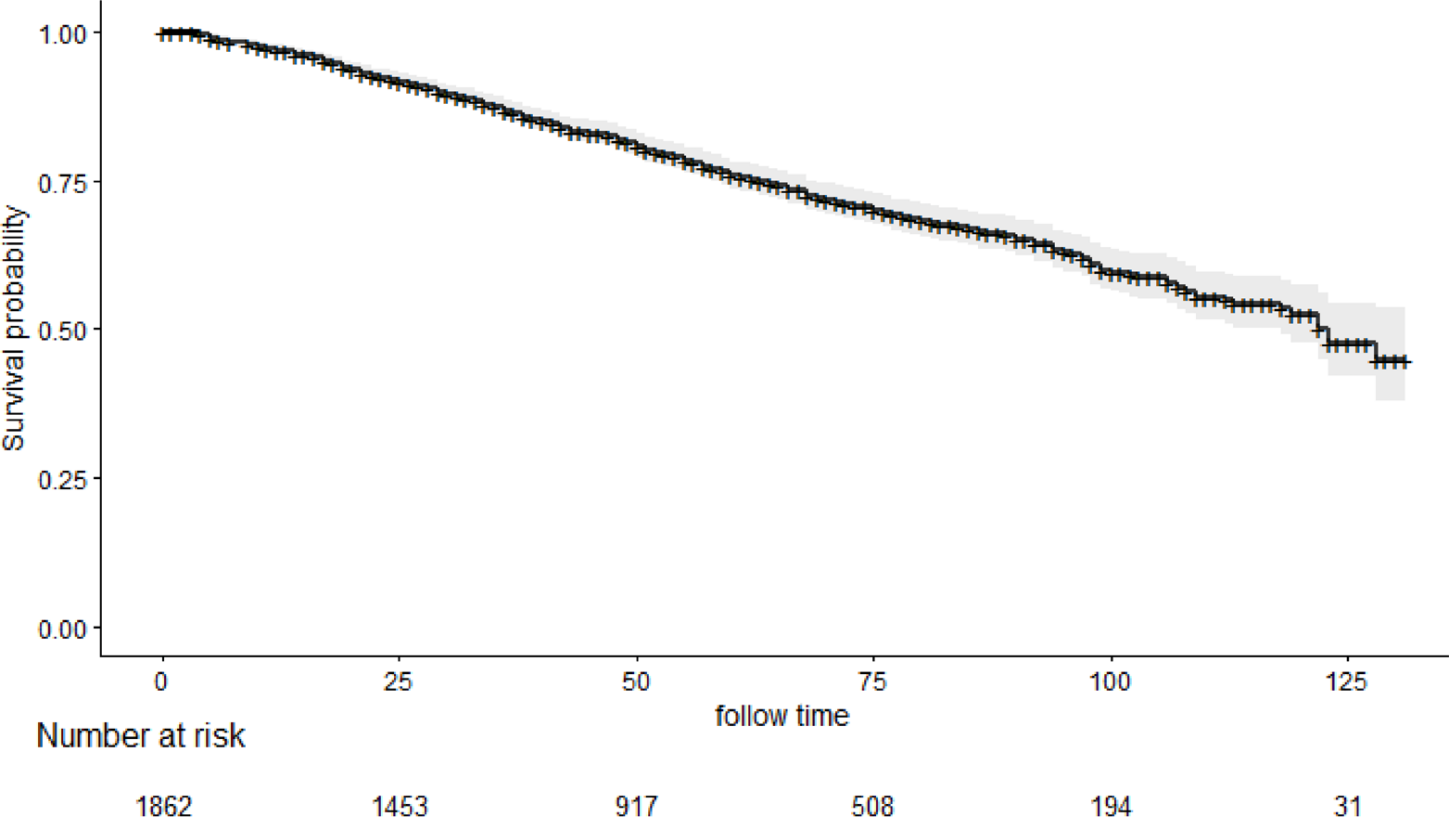
Kaplan–Meier survival curves of overall survival (OS) in all included male breast cancer.

In total, 1862 eligible MBC patients with IDC type diagnosed from January 1, 2004 to December 31, 2013 were enrolled in our study. The specific screening process was shown in Fig. 1. Among them, 1,174 and 688 patients were in the training cohort and validation cohort, respectively. The median follow-up time was 49 months (range: 0–131 months). Median age at diagnosis was 65 years (27–97 years).The 3- and 5-year OS rates were 86.76% and 75.80%, respectively. The OS curve of all included MBC patients is shown in Fig. 2. Among them, 67.78% of patients were married. The most common primary site was the central portion (41.41%). Receptor positivity was detected, with estrogen positive in 96.94% and progesterone positive in 88.51% of cases. Additional irradiation was performed in 26.64% of patients, and chemotherapy was conducted in 43.66% of patients. Except for primary site (*P* < 0.001), the other 14 variables were not significantly different between the two groups. The demographic and clinicopathological traits are shown in Table 1.

**Table 1:** The demographics and pathological characteristics of included patients.

Variables	All patients (*n* = 1, 862)	Training set (*n* = 1, 174)	Validation set (*n* = 688)	*P*-value^a^
	N (%)	N (%)	N (%)	
Age				0.996
>20 and ≤45	104 (5.59%)	66 (5.62%)	38 (5.52%)	
>45 and ≤70	1,131 (60.74%)	713 (60.73%)	418 (60.76%)	
>70	627 (33.67%)	395 (33.65%)	232 (33.72%)	
Race				0.561
white	1,512 (81.20%)	959 (81.69%)	553 (80.38%)	
black	240 (12.89%)	144 (12.27%)	96 (13.95%)	
other	110 (5.91%)	71 (6.05%)	39 (5.67%)	
Marital status				0.233
married	1262 (67.78%)	803 (68.40%)	459 (66.72%)	
unmarried	512 (27.50%)	323 (27.51%)	189 (27.47%)	
unknown	88 (4.73%)	48 (4.09%)	40 (5.81%)	
Primary site				<0.001
Other site	901(48.39%)	603 (51.36%)	298 (43.31%)	
upper-inner quadrant	68 (3.65%)	35 (2.98%)	33 (4.80%)	
lower-inner quadrant	31 (1.66%)	14 (1.19%)	17 (2.47%)	
upper-outer quadrant	209 (11.22%)	141 (12.01%)	68 (9.88%)	
lower-outer quadrant	68 (3.65%)	34 (2.90%)	34 (4.94%)	
overlapping lesion	248 (13.32%)	167 (14.22%)	81 (11.77%)	
breast, NOS	337 (18.10%)	180 (15.33%)	157 (22.82%)	
Laterality				0.297
left	982 (52.74%)	630 (53.66%)	352 (51.16%)	
right	880 (47.26%)	544 (46.34%)	336 (48.84%)	
Grade				0.914
Grade1+2	1164 (62.51%)	735 (62.61%)	429 (62.35%)	
Grade3+4	698(37.49%)	439 (37.39%)	439 (37.39%)	
T stage				0.109
T1	890 (47.80%)	538 (45.83%)	352 (51.16%)	
T2	786 (42.21%)	520 (44.29%)	266 (38.66%)	
T3	50 (2.69%)	30 (2.56%)	20 (2.91%)	
T4	136 (7.30%)	86 (7.33%)	50 (7.27%)	
N stage				0.546
N0	983 (52.79%)	613 (52.21%)	370 (53.78%)	
N1	581 (31.20%)	379 (32.28%)	202 (29.36%)	
N2	190 (10.20%)	118 (10.05%)	72 (10.47%)	
N3	108 (5.80%)	64 (5.45%)	44 (6.40%)	
M stage				0.676
M0	1787 (95.97%)	1125 (95.83%)	662 (96.22%)	
M1	75 (4.03%)	49 (4.17%)	26 (3.78%)	
Tumor size				0.048
≤2	908 (48.76%)	547 (46.59%)	361 (52.47%)	
>2 and ≤5	878 (47.15%)	578 (49.23%)	300 (43.60%)	
>5	76 (4.08%)	49 (4.17%)	27 (3.92%)	
ER				0.566
negative	57 (3.06%)	38 (3.24%)	19 (2.76%)	
positive	1805 (96.94%)	1136 (96.76%)	669 (97.24%)	
PR				0.540
negative	214 (11.49%)	139 (11.84%)	75 (10.90%)	
positive	1648 (88.51%)	1035 (88.16%)	613 (89.10%)	
HER-2				0.214
negative	738 (39.63%)	480 (40.89%)	258 (37.50%)	
positive	123 (6.61%)	81 (6.90%)	42 (6.10%)	
unknown	1001 (53.76%)	613 (52.21%)	388 (56.40%)	
Chemotherapy				0.523
no/unknown	1049 (56.34%)	668 (56.90%)	381 (55.38%)	
yes	813 (43.66%)	506 (43.10%)	307 (44.62%)	
Radiotherapy				0.534
no/unknown	1366 (73.36%)	867 (73.85%)	499 (72.53%)	
yes	496 (26.64%)	307 (26.15%)	189 (27.47%)	

**Notes.**

a The comparison results between Training set and Validation set.

### Nomogram construction

The factors independently and significantly influencing the OS in the multivariate analysis are shown in Table 2. After adjustment of other risk factors, eight variables were revealed as independent predictive factors, including: age (*P* < 0.0001), marital status (*P* = 0.002), T stage (*P* < 0.0001), N stage (*P* = 0.021), M stage (*P* < 0.0001), PR (*P* = 0.046), HER2 (*P* = 0.009), and chemotherapy (*P* = 0.003).

**Table 2:** Univariate and multivariate analyses of overall survival in the training set.

Variables	Univariate analysis			Multivariate analysis	
	HR(95% CI)	*P*-value		HR(95% CI)	*P*-value
Age		<0.0001			<0.0001
>20 and ≤45	Reference			Reference	
>45 and ≤70	1.190(0.625, 2.267)	0.595		0.989(0.513–1.909)	0.974
>70	3.225 (1.702, 6.111)	<0.0001		2.669(1.376,5.176)	0.004
Race		0.007		–	
white	Reference				
black	1.458 (1.063, 2.000)	0.019			
other	0.571 (0.320, 1.020)	0.058			
Marital status		<0.0001			0.002
married	Reference			Reference	
unmarried	1.998 (1.573, 2.538)	<0.0001		1.551(1.208,1.992)	0.001
unknown	1.517 (0.823, 2.794)	0.181		1.370(0.733–2.561)	0.324
Primary site		0.050		–	
other site	Reference				
upper-inner quadrant	0.832 (0.494, 1.402)	0.491			
lower-inner quadrant	0.675(0.278, 1.636)	0.384			
upper-outer quadrant	0.709 (0.506, 0.995)	0.046			
lower-outer quadrant	0.569 (0.302, 1.071)	0.081			
overlapping lesion	0.702 (0.513, 0.963)	0.028			
breast, NOS	1.059 (0.840, 1.335)	0.629			
Laterality				–	
left	Reference				
right	0.884 (0.703, 1.113)	0.294			
Grade				–	
Grade1+2	Reference				
Grade3+4	1.679 (1.398, 2.017)	<0.0001			
T stage		<0.0001			<0.0001
T1	Reference			Reference	
T2	2.763 (2.107, 3.623)	<0.0001		2.417(1.829, 3.194)	<0.0001
T3	4.913 (2.820, 8.560)	<0.0001		2.966(1.579, 5.573)	0.0001
T4	4.436 (3.024, 6.508)	<0.0001		2.958(1.923,4.549)	<0.0001
N stage		<0.0001			0.021
N0	Reference			Reference	
N1	1.388 (1.069, 1.802)	0.013		1.404(1.066,1.849)	0.016
N2	1.724 (1.206, 2.464)	0.002		1.673(1.121,2.498)	0.012
N3	2.200 (1.476, 3.281)	0.0001		1.645(1.049,2.578)	0.030
M stage					
M0	Reference			Reference	
M1	5.336 (3.713, 7.668)	<0.0001		3.661(2.376,5.641)	<0.0001
Tumor size		<0.0001		–	
≤2	Reference				
>2 and ≤5	2.790 (2.150, 3.621)	<0.0001			
>5	5.225 (3.330, 8.197)	<0.0001			
ER				–	
negative	Reference				
positive	0.710 (0.406, 1.239)	0.228			
PR					
negative	Reference			Reference	
positive	0.736 (0.541, 1.001)	0.050		0.720 (0.522,0.994)	0.046
HER-2		0.035			0.009
negative	Reference			Reference	
positive	1.941 (1.104, 3.413)	0.021		2.344 (1.318,4.167)	0.004
unknown	1.410 (1.015, 1.960)	0.041		1.431 (1.026,1.996)	0.035
Chemotherapy					
no/unknown	Reference			Reference	
yes	0.746 (0.591, 0.942)	0.014		0.651 (0.490,0.864)	0.003
Radiotherapy				–	
no/unknown	Reference				
yes	1.071 (0.832, 1.379)	0.592			

**Figure 3:**
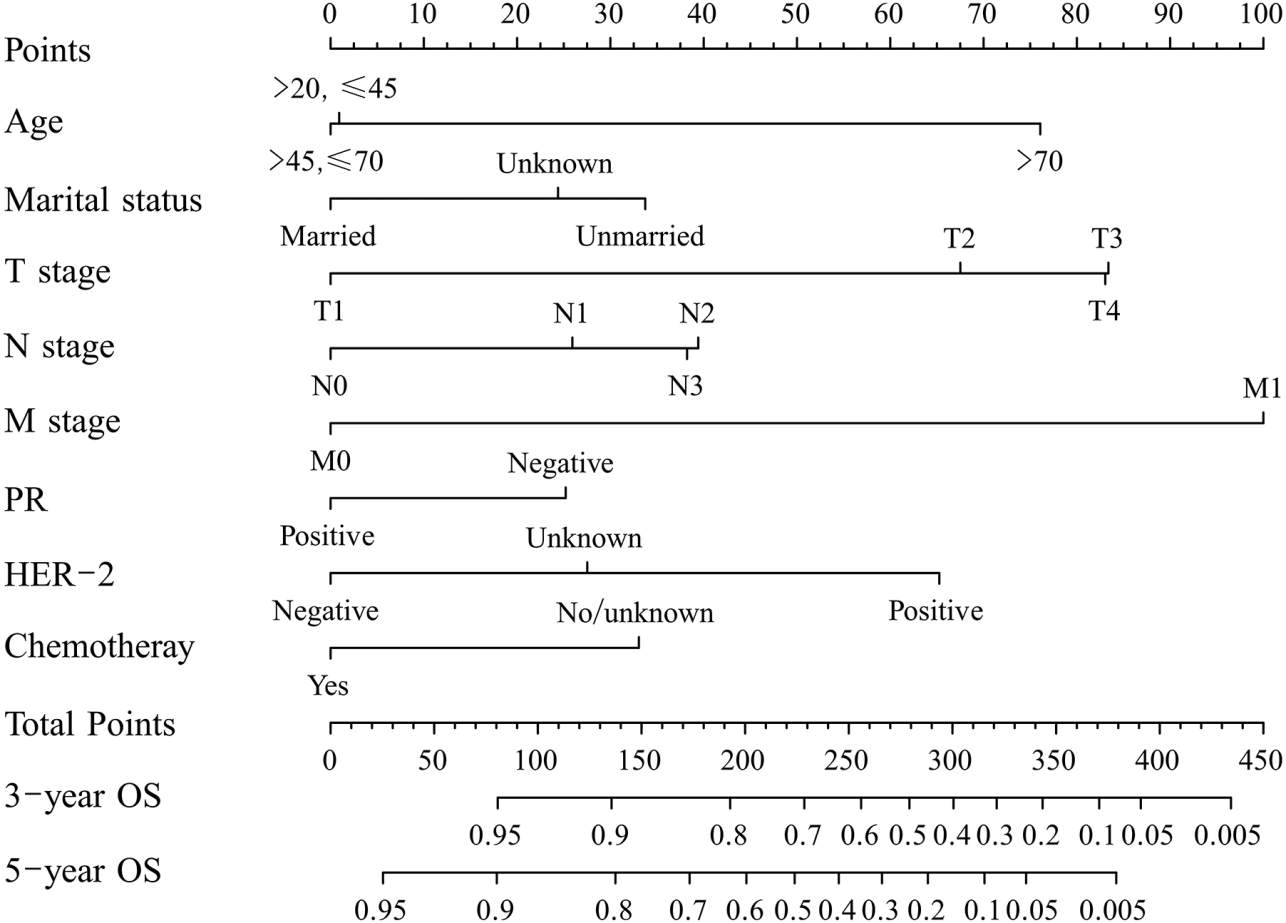
A nomogram for predicting 3- and 5-year overall survival (OS) of patients with MBC. The nomogram is used by summing the points identified on the top scale for each independent variable and drawing a vertical line from the total points scale to the 3- and 5-year OS to obtain the probability of survival. The total points projected to the bottom scale indicate the % probability of the 3- and 5-year survival.

A nomogram to predict 3- and 5-year OS was established on the basis of independent variables in the training cohort (Fig. 3). It demonstrated that M stage made the greatest contribution to prognosis, followed by T stage, age, HER2, N stage, marital status, chemotherapy, and PR. The addition of the scores of all selected variables gave rise to the easy calculation of the survival possibility of individual patient.

### Nomogram validation

**Figure 4:**
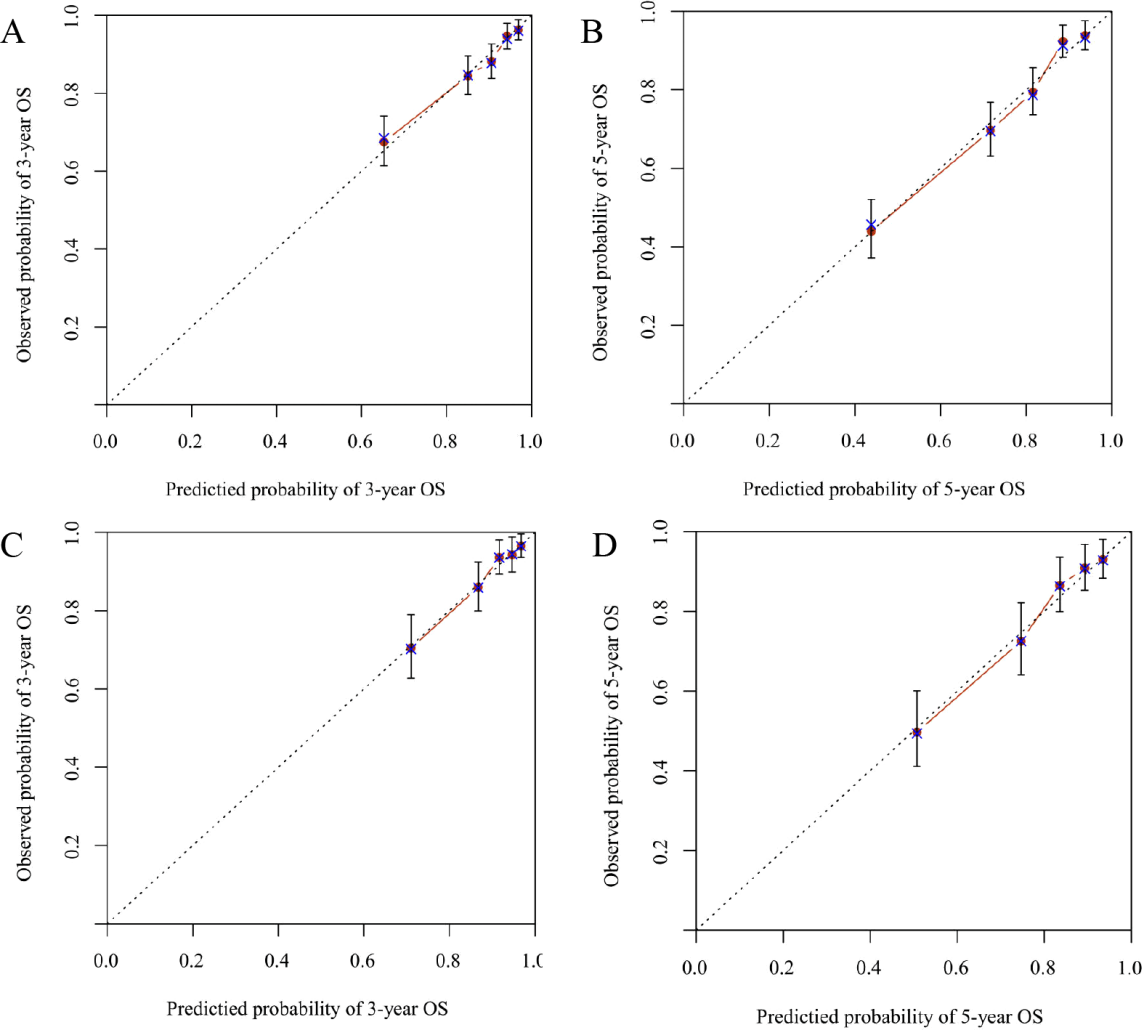
Calibration plots of the nomogram for 3-and 5-year overall survival (OS) (A, B) prediction in the training set, and 3-and 5-year OS (C, D) prediction in the validation set. The *X*-axis represents the nomogram-predicted probability of survival; the *Y*-axis represents the actual OS probability. Plots along the 45-degree line indicate a perfect calibration model in which the predicted probabilities are identical to the actual outcomes. Vertical bars indicate 95% confidence intervals.

**Figure 5:**
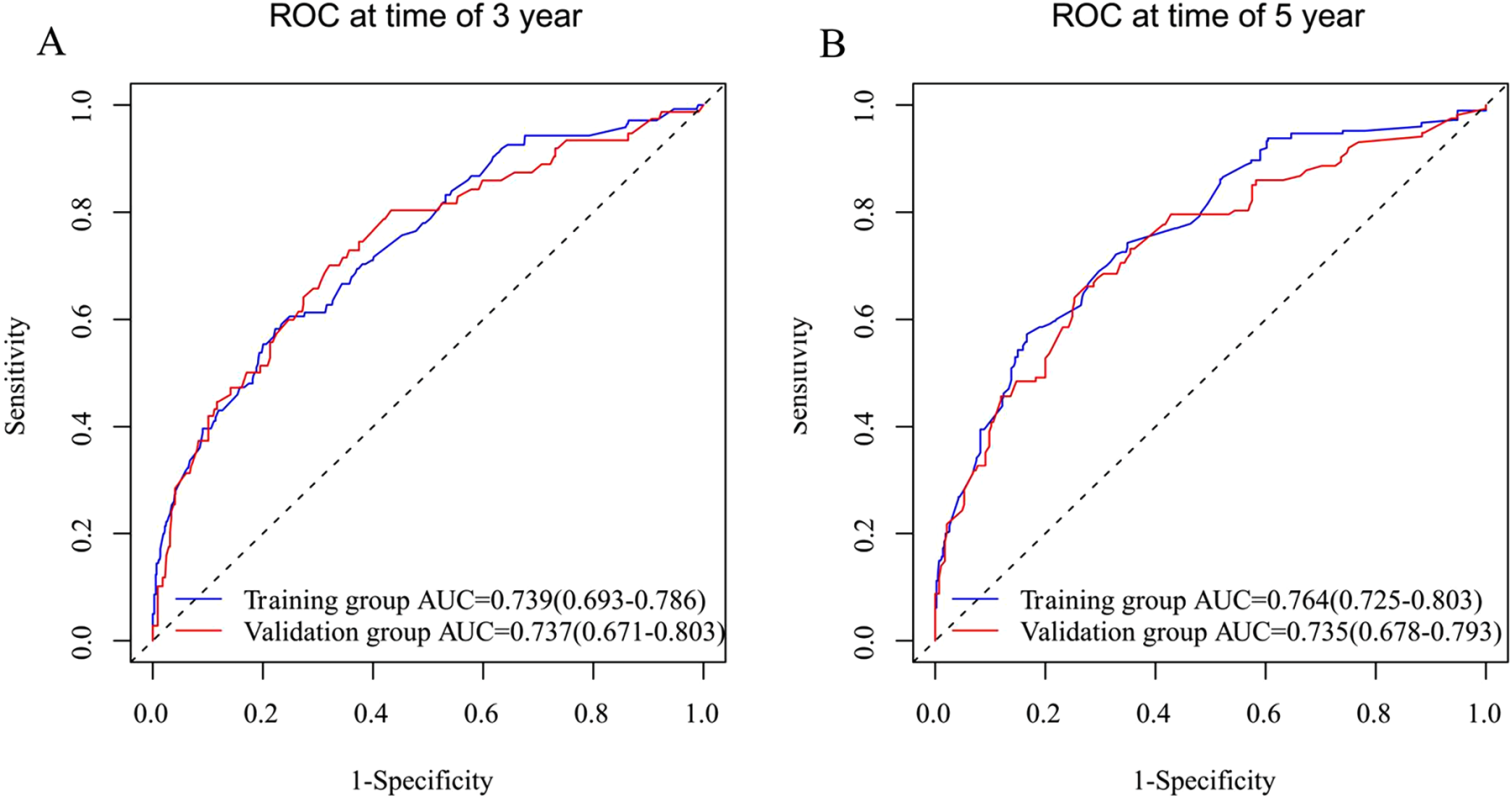
Discriminatory accuracy for predicting OS assessed by receiver operator characteristics (ROC) analysis calculating area under the curve (AUC). Three-year OS in the training and validation cohort (A). Five-year OS in the training and validation cohort (B).

Both internal and external validations of the nomogram were performed. On one hand, internal validation from the training set revealed that the C-index for OS prediction in the nomogram was 0.740 (95% CI [0.709–0.771]). On the other hand, external validation from the validation set demonstrated that the C-index for OS prediction in the nomogram was 0.718 (95% CI [0.672–0.764]). Furthermore, the discriminatory capacity of the nomogram was compared with that of SEER stage and TNM 6th staging classification. Consequently, the discriminatory power for OS prediction was significantly superior in the nomogram compared to that in the SEER or TNM staging classification in training and validation sets (*P* < 0.001) (Table 3). Moreover, good agreement was detected between the nomogram predictions and actual observation through the internal and external calibration plots (Fig. 4). The associated ROC of the training and validation cohort was shown in Fig. 5. The area under the curve (AUC) for 3-year OS was 0.739 (95% CI [0.693–0.786]) in the training cohort and 0.737 (95% CI [0.671–0.803]) in the validation cohort. The AUC for 5-year OS was 0.764 (95% CI [0.725–0.803]) in the training cohort and 0.735 (95% CI [0.678–0.793]) in the validation cohort.

**Table 3:** C-indexes for the nomograms and other stage systems in patients with MBC.

Survival		Training set			Validation set	
		C-index(95% CI)	*P*-Value^a^		C-index(95% CI)	*P*-Value^a^
OS	Nomogram	0.740(0.709, 0.771)			0.718(0.672, 0.764)	
	AJCC 6th stage	0.659(0.624, 0.694)	<0.001		0.638(0.591, 0.685)	<0.001
	SEER summary stage	0.609(0.577, 0.641)	<0.001		0.605(0.561, 0.649)	<0.001

**Notes.**

a All are compared with Nomogram.

OS overall survival HR hazard ratio CI confidence interval

**Figure 6:**
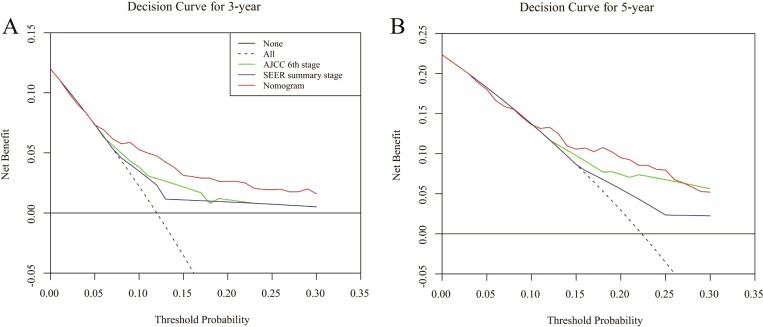
Decision curve analysis for the Nomogram, AJCC 6th stage and SEER summary stage in prediction of prognosis of male patients at 3-year (A) and 5-year (B) point in the validation cohorts.

DCA was performed to compare the clinical usability and benefits of the nomogram with that of the traditional AJCC 6th stage and SEER summary stage. As shown in Fig. 6, compared to the AJCC stage and SEER summary stage model, the new nomogram’s 3- and 5-year DCA curves showed larger net benefits across a range of death risks in the validation cohort.

## Discussion

We aimed to establish and confirm a prognostic nomogram for OS prediction of MBC with IDC histology. In total, 1,862 MBC patients with IDC histology were extracted from the SEER dataset for analysis. We successfully constructed a nomogram for 3- and 5-year OS prediction in MBC with IDC histology, which was confirmed by the favorable discrimination and calibration in both internal and external validations. Moreover, more potent predictive capacity was observed in the nomogram compared with the SEER stage and TNM staging classification.

At the present time, the treatment of MBC is based on the guidelines developed for FBC (Rizzolo et al., 2013). However, it is known that FBC and MBC differ biologically. Many scholars found that the levels of hormone receptors in malignant tumors of the male mammary gland were higher than those in malignant female breast tumors on average. The presence of receptor-positive tumors in men does not increase with the age, as is observed in FBC (Gucalp et al., 2019). The results are basically consistent with our research. Our study found that the positive rate of estrogen receptor was 96.94%, and that of progesterone receptor was 88.51%.

Common FBC risk factors such as age, hormone receptor status, stage, and therapy are also involved in the prognosis of MBC. Our model consisted of eight independent prognostic indicators such as age, marital status, T stage, N stage, M stage, PR, HER2, and chemotherapy. Age has been revealed as a critical prognostic indicator for OS in several studies (Brinton et al., 2014; Oger et al., 2015). Consistently, we found that patients over 70 years harbor a lower OS than the younger patients. Moreover, the mortality risk of unmarried MBC patients is significantly increased compared to married populations, despite the undefined mechanism by which this occurs (Liu et al., 2018).

MBC is highly likely to have estrogen and progesterone receptors (Bezwoda et al., 1987), indicating that endocrine factors might also be critically involved in pathogenesis. More recently, various other receptors have been discovered in MBC, including HER2, androgen, and epidermal growth factor receptor (EGFR), in spite of the unclear understanding of the prognostic significance of these receptors (Ravandi-Kashani & Hayes, 1998). In our study, we found that PR negative and HER2 positive are independent unfavorable prognostic factors.

Although chemotherapy data in MBC originate from small, nonrandomized clinical studies, adjuvant chemotherapy seems to decrease recurrence and mortality risks in MBC (Giordano et al., 2005; Walshe et al., 2007). To be specific, in a study enrolling 135 cases of MBC (Giordano et al., 2005), 62% of them underwent adjuvant chemotherapy (with or without endocrine therapy),which was related to a decreased trend of mortality for node-positive patients. Similarly, we also found that chemotherapy was an independent protective prognostic indicator (HR: 0.651; 95% CI [0.490–0.864]).

Nomograms can be used as statistical tools for providing survival possibility of specific outcomes through a simple graphical presentation (Balachandran et al., 2015). Moreover, nomograms have been validated with a superior predictive capacity than the classic TNM staging classification in certain types of malignancies, which therefore has been characterized as an alternative and novel standard (Bagaria et al., 2015; Cao et al., 2016). Moreover, nomograms are especially appropriate to deal with complicated situations without the presence of standard clinical guidelines (Lin et al., 2001; Sheu et al., 2014).

To the best of our knowledge, this study is the first one to explore the use of a nomogram to specially predict the individualized postoperative survival of IDC type of MBC, which can provide opportunities for clinicians to classify the patients according to risk scores and help select therapeutic strategies. Moreover, superior discriminatory capacity was observed in our nomogram compared to the SEER or TNM staging classification, with respect to OS prediction. Two other studies have also established prognostic nomograms for MBC (Sun et al., 2019; Wang et al., 2018). Sun et al. (2019) established a nomogram for predicting breast cancer-specific death and other cause-specific deaths of non-metastatic MBC. Compared with our study, the population and the endpoints were not the same. Wang et al. also established a nomogram for predicting the OS of early breast cancer patients (T_1−2_N_0−2_M_0_). They found that age, marital status, grade, T stage, N stage, ER, surgery, chemotherapy, and radiation therapy were independent prognostic factors of OS (Wang et al., 2018). The results of their study are basically consistent with our findings; however, there are still differences between the two studies in the patients included.

There were some limitations in our study. First, although eight variables were involved, there are still some variables that SEER does not include, such as family history, surgical margin status, and vascular invasion. Second, selection bias might exist as we only included patients with complete information of involved variables. Third, we only analyzed MBC patients with histology of IDC. Other types of MBC were not analyzed, including medullary lesions, infiltrating lobular carcinoma, tubular, or neuroendocrine tumors.

## Conclusion

In conclusion, we constructed and validated a nomogram in patients with IDC type of MBC after surgery based on the SEER database. The proposed nomogram can be widely and easily used in clinical practice, which facilitates the prevalence of patient counseling as well as individualized therapy. However, it is necessary to further reinforce the unknown prognostic factors to optimize the nomogram, and more external validation is still needed.

##  Supplemental Information

10.7717/peerj.7837/supp-1 Data S1 Raw data: included patients

## Abbreviation

MBC: male breast cancer
FBC: female breast cancer
IDC: infiltrating duct carcinoma
OS: overall survival
SEER: Surveillance, Epidemiology, and End Results
C-index: concordance index
AJCC: American Joint Committee on Cancer
PH: proportional hazard
CI: confidence interval
HR: hazard ratio

## Additional Information and Declarations

### Data Availability

The following information was supplied regarding data availability: The raw data is available as a Supplemental File.
